# A Family of Opium Lovers

**Published:** 1854-12

**Authors:** 


A Family of Opium Lovers.—Some six months ago, a person visited our town, asking money to purchase medicines for his mother, who was sick. Recently, the same solicitor has been around on the same errand for other members of the family. What success has attended his solicitations, we know not; but the object to which the funds are applied, is of so objectionable a nature that all should withhold their names, out of regard for the family, who are the slaves of a habit to which drunkenness the most degrading is a comparative blessing. The entire family, it is said, subsist for the most part on opium, or its exhilarating and soporific influence, and this fearful habit has been so long indulged as to have grown into a second life. The example of Coleridge before the world, who acknowledged it the basest and most destructive of vices, and at the same time the most absolute of tyrants should, we think, be a sufficient warning to all after-comers to avoid the drug; but here is an example of a whole family addicted to the habit; and it has brought upon them all its certain results—apathy, indolence, poverty, misery—and will eventually end in the most wretched death.—*Elmira Republican.*

